# The environmental impact of religious beliefs in the East and West: evidence from China

**DOI:** 10.3389/fpsyg.2024.1432142

**Published:** 2024-12-23

**Authors:** Junyan Yang, Chuntian Lu

**Affiliations:** Institute for Empirical Social Science Research (IESSR), Department of Sociology, School of Humanities and Social Sciences, Xi'an Jiaotong University, Xi'an, China

**Keywords:** religion, environment, nature empathy, anthropocentrism, SEM model

## Abstract

This study explores the influence of religious beliefs on environmental attitudes and behaviors in China. Using data from the 2021 Chinese General Social Survey (CGSS), two structural equation models were constructed to examine the mediating roles of natural empathy and anthropocentrism in the relationship between environmental awareness and willingness to make sacrifices for environmental protection. The results indicated that while environmental awareness positively influenced willingness to sacrifice, natural empathy did not significantly mediate this relationship. Conversely, anthropocentrism negatively mediated the relationship, suggesting that individuals with anthropocentric tendencies were less willing to make personal sacrifices for environmental protection. Furthermore, a multi-group analysis revealed that individuals with traditional Eastern religious beliefs (Buddhism, Taoism, and folklore) exhibited higher environmental awareness and willingness to sacrifice compared to those with no religion or traditional Western (Christianity) religious beliefs. These findings highlight the influence of religious traditions, particularly the emphasis on nature reverence in Eastern religions, on shaping pro-environmental attitudes and behaviors. The study contributes to understanding the complex interplay between religious beliefs, environmental values, and sustainable behaviors in the Chinese context.

## 1 Introduction

The global environmental crisis represents one of the most pressing challenges of our time, threatening the sustainability of both ecological systems and human societies. Effectively addressing these challenges requires a multifaceted approach that goes beyond technological and policy solutions to include cultural and belief systems that shape human attitudes and behaviors toward nature. Among these cultural factors, religion—an enduring and influential tradition—plays a pivotal role in shaping societal norms, regulating polity and economy, and guiding individual life processes (Hekmatpour, 2020; Williamson, 2000). It profoundly influences the goals, activities, mindsets, and behaviors of its adherents (Ip, 2009), including how individuals and communities perceive and interact with the natural environment. Studies examining the interplay between environmental change and religion have highlighted how climate change influences religious perspectives (Clingerman and O'Brien, 2017; Haluza-DeLay, 2014; Jenkins et al., 2018). Fieldwork-based case studies further demonstrate that diverse religious practices, such as reverence for nature and prioritization of environmental issues, shape people's attitudes and behaviors toward the environment (Allen, 2018; Koehrsen, 2021). Moreover, religious organizations, particularly supra-local incumbent institutions, have been identified as significant actors in advancing environmental protection efforts (Koehrsen and Huber, 2021).

Existing research on the relationship between religion and environmental concern has yielded complex and often contradictory findings (Pudlo, 2019). While some studies suggest that certain religious beliefs, particularly those rooted in Western traditions like Christianity, may promote anthropocentric worldviews and hinder environmental concern (Boyd, 1999; White, 1967), others have found positive associations between religiosity and pro-environmental attitudes and behaviors (Habito, 2007; Sherkat and Ellison, 2007). Simultaneously, a group of researchers have undertaken an investigation into the question of whether religions are displaying a growing proclivity toward environmentally sustainable practices, through a comprehensive review of the abundant literature on the subject (Taylor et al., 2016). They believe that the relationship between religious beliefs and environmental concerns and behaviors cannot be generalized. These inconsistencies highlight the need for a more nuanced understanding of the underlying mechanisms through which religious beliefs influence environmental attitudes and behaviors.

This study explores how natural empathy and anthropocentrism mediate the relationship between environmental awareness and willingness to make sacrifices for environmental protection. We examine how religious beliefs influence individuals' empathy toward nature and prioritization of human interests, thereby shaping pro-environmental behaviors. Focusing on China's diverse religious traditions, we investigate how Eastern religions, such as Buddhism and Taoism, with their emphasis on harmony with nature, differ from Western religions in shaping environmental attitudes and behaviors. Using data from the 2021 Chinese General Social Survey (CGSS) and structural equation modeling, this study aims to clarify the interplay between religious beliefs, natural empathy, anthropocentrism, and environmental concern in the Chinese context.

## 2 Literature review

### 2.1 Christianity and anthropocentrism

Western religions, particularly Christianity, are often critiqued for their “anthropocentric” worldview. White (1967) had already argued that the environmental crisis we face has deep roots in Western Christian thought. He critiques the Judeo-Christian concept of humans having “dominion” over creation. He posits that radical transformations in religious cosmologies are indispensable if we aim to halt or even reverse the anthropogenic harm to the environment (White, 1967). James M. Gustafson, however, offered a more nuanced perspective in *A Sense of the Divine: The Natural Environment from a Theocentric Perspective*. He acknowledged that Christian theology includes both anthropocentric and theocentric elements. While Genesis 1:28 has been interpreted to justify human domination, other biblical texts, such as Psalm 24:1–2, emphasize that “the earth is the Lord's” and humans are merely stewards of creation (Gustafson, 1994). Gustafson argued that placing God, rather than humanity, at the center of environmental ethics could foster a sense of responsibility and care for the natural world, countering exploitative tendencies.

Empirical research highlights the complexity of these theological interpretations in practice. Research indicated a correlation between anthropocentrism and religion, particularly in relation to measures of doctrinal orthodoxy and religious orientation (Snodgrass and Gates, 1998). A study conducted by Boyd (1999) found that when examined without considering other religion variables, only membership in a fundamentalist tradition was weakly associated with support for the environment. There are also differences between different denominations; fundamentalist Christians were found to be less pro-environment than moderate and liberal Christians (Amster, 2008; Arbuckle and Konisky, 2015).

However, environmental scholars have pointed out that White's focus on Christianity simplifies the issue. In the United States, for example, some researches have reported that due to the complex interactions between religious beliefs, political orientation, and environmental concern, the connection between religion and environmental concern shows a positive relationship (Arbuckle, 2017; Sherkat and Ellison, 2007). In recent years, efforts have emerged within Christianity to promote environmental stewardship. The Christian environmental movement, as documented by Taylor and his team seeks to integrate sustainability into faith-based practices (Taylor et al., 2016). Nevertheless, its impact remains limited. Another research found no significant increase in pro-environmental behaviors among average Christians, suggesting that such efforts have not yet achieved widespread cultural change (Clements et al., 2014). Furthermore, Carlisle and Clark (2018) concluded that shifts in environmental concern are better explained by broader societal changes than by denominational shifts, underscoring the limited reach of the “greening” of Christianity.

We argue that, with an anthropocentric religious ideology and a lack of pro-environmental practices in their traditions, Christians exhibit an indifference and neglect toward the environment which differentiates them from deliberate environmental devastation. Some studies have also demonstrated this phenomenon, they have shown that religious beliefs do not have a significant effect on environmental attitudes (Hayes and Marangudakis, 2000), and Christians do not show strong concern for the environment (Konisky, 2018; Peifer et al., 2014).

Overall, while Christianity's anthropocentric interpretations have historically shaped environmental attitudes, ongoing debates and theological developments reveal its potential to contribute to environmental ethics. The contrast between White's critique and Gustafson's theocentric framework illustrates the dual capacity of religious thought to hinder or foster ecological awareness, depending on its interpretation and application.

### 2.2 Eastern religious traditions and nature empathy

Lynn White's analysis of Christianity's role in the ecological crisis has been critiqued for overlooking how other religious traditions engage with the environment. Many religious systems, particularly those rooted in Eastern and Indigenous traditions, emphasize stewardship, interconnectedness, and harmony with nature. Studies have shown that adherents of Eastern religions, such as Hinduism, Buddhism, and Taoism, often demonstrate stronger pro-environmental behaviors compared to their Western counterparts (Habito, 2007; Lai et al., 2022; Liu, 2015). This is largely attributed to the teachings of these traditions, which deeply revere nature and promote balance between humanity and the environment. Many Eastern spiritual practices incorporate mindfulness techniques that encourage individuals to appreciate the intricate connections between all living things on Earth (Bhatia et al., 2017). Practices such as mindfulness and animistic reverence for spirits in nature further reinforce these ecological values, fostering a culture of environmental care among their followers.

Empathy for nature is a key factor in explaining the pro-environmental inclinations of individuals influenced by Eastern religious beliefs. Past research by Marina Ienna and her consortium showed that the ability to empathize with nature can be used to assess people's pro-environmental attitudes and behaviors (Ienna et al., 2022). Another study exploring the relationship between gender and environmentalism, natural empathy was considered as a mediating variable, providing an explanation for why women appear to display greater concern for the environment than men (Arnocky and Stroink, 2010). Empathy is considered to have a significant impact on people's environmental attitudes and behaviors. To further examine the correlation between empathy toward nature and environmental preservation, psychologists Kim-Pong Tam makes the process of measuring individuals' empathy toward the natural environment (specifically animals and plants) by creating and testing the Dispositional Empathy with Nature Scale (DENS), and investigating its potential impact on pro-environmental actions (Tam, 2013). Social psychologist Berenguer believes that empathy, moral reasoning, and pro-environmental behaviors are interconnected, and empathy acts as a bridge between emotional connection to the environment and taking action to protect it (Berenguer, 2007, 2010).

Environmental empathy also plays an important role in explaining the relationship between Eastern religious beliefs and environmental protection behavior. Eastern religious beliefs have long been esteemed for their emphasis on respecting nature and fostering harmony between heaven and humanity. For instance, Buddhism places a strong emphasis on the interconnectedness of all things, encompassing humans and the natural world. Buddhist teachings highlight the significance of non-harm and compassion toward all living beings, including animals and plants (Habito, 2007). This religious doctrine has fostered numerous pro-environmental traditional practices, continually shaping the environmental behaviors of its followers. For example, Darlington examined how one northern Thai monk utilized a tree ordination, adapted from a traditional Buddhist ritual, to foster villagers' commitment to his ecology projects (Darlington, 1998). Saloni Bhatia and their team investigated how the concept of “human-wildlife interdependence” in Buddhist beliefs influences Indian attitudes toward wild large carnivores (Bhatia et al., 2017).

Taoism, as the most prominent indigenous religion in China, has also significantly influenced societal attitudes toward environmental protection (Kroll et al., 1997). Central to Taoist philosophy is the concept of the “unity of nature and man,” which advocates harmonious coexistence with the natural world (Lai et al., 2022). Taoism exerted influence on people's awareness and behaviors in environmental protection (Wang and Stringer, 2000). For example, Taoist teachings encourage low-carbon lifestyles (Zhang et al., 2022), sustainable development and environmental conservation (Lim, 1990).

Given the diverse ecological teachings embedded in both Eastern and Western religious traditions, this study utilizes a dataset from China to explore the multifaceted relationship between religious ideologies and environmental behaviors.

## 3 Research methodology

### 3.1 Data

In this study, we using the CGSS 2021 dataset (Chinese General Social Survey). CGSS is a continuous cross-sectional survey dataset covering mainland China. It gathers data at various levels of Chinese society, including communities, households, and individuals, using a multi-stage stratified sampling approach.

The CGSS 2021 dataset is both systematic and comprehensive, incorporating surveys on religious beliefs and environmental issues (http://www.cnsda.org/index.php?r=projects/view&id=65635422). This dataset includes an environmental module with several items that are used to construct measures of anthropocentrism, nature empathy, attitudes toward environmental protection, and behavior. For this study, we selected a subset of the full database consisting of respondents who had provided information on both their religious beliefs and their attitudes and behaviors related to environmental protection. After data screening, the valid sample size for this study was 1,860.

The inclusion of both religious belief and environmental issue modules in the CGSS provides a unique opportunity to explore the relationship between religious views and environmental protection attitudes across a representative national sample. Additionally, the dataset includes demographic information, which allows for controlling potential confounding factors and strengthens the validity of the analysis.

### 3.2 Variables

We create two variables: “Empathy with Nature” (EN) and “Anthropocentrism” (AM). To measure these two variables, we referred to previous studies. For the Empathy with Nature (EN), we referred to the DENS measure (Tam, 2013), which measures and calculates people's level of nature empathy through their attitudes and feelings toward the natural environment. For the Anthropocentrism (AM), we have taken into consideration the Anthropocentrism Scale utilized in psychology (Snodgrass and Gates, 1998). By referencing items from this scale, we handpicked questions from the CGSS questionnaire that also sufficiently expressed the anthropocentric tendencies of respondents. Further, we computed the resulting data.

Moreover, we developed composite indicators to assess two aspects of environmental protection, include attitudes and behaviors:

a. Awareness of environmental consequences (AC): perceive that life has environmental problems needs to be protected;
b. Willingness to sacrifice for the environment (WE): the extent to people who is willing to pay or make sacrifices for the environment.

Given that these two variables were directly measured in the CGSS questionnaire, we are able to utilize them straightaway. Following is the list of questionnaire items relevant to the variables under study (Table 1):

- Firstly, to address the reverse-coded questions in the questionnaire, we conducted secondary coding, recoding responses of “strongly disagree” to “strongly agree” and vice versa.
- Secondly, we utilized factor score weights from Confirmatory Factor Analysis (CFA) to create composite indicators for four constructs: “Anthropocentrism” “Empathy with Nature” “Awareness of Environmental Consequences” and “Willingness to Sacrifice for the Environment.”

**Table 1:** Questionnaire items related to variables.

**Empathy with nature (five-point Likert scale)**	
H3	Human destruction of nature often leads to catastrophic consequences
H5	Humans are currently abusing and destroying the environment
H13	The balance of nature is very fragile and can be easily disturbed
**Anthropocentrism (five-point Likert scale)**	
H2	People are the most important and can change the natural environment to meet their own needs
H6	The Earth's natural resources are plentiful if we know how to exploit them
H8	Nature's ability to balance itself is strong enough to cope with modern industrial society
H12	Humans are born masters and are meant to rule over the rest of the natural world
H14	Humans will eventually know more about the laws of nature and thus have the ability to control it
**Awareness of environmental consequences**	
**(five-point Likert scale)**	
P2	Even if it costs more money and time, I want to do what is good for the environment
P7	Environmental issues directly affect my daily life
P3a	There are more important things to do in life than to protect the environment
P4a	Unless everyone does it, my efforts to protect the environment will be meaningless
P5a	Many claims of environmental threats are exaggerated
**Willingness to sacrifice for the environment**	
**(five-point Likert scale)**	
L1a	To what extent are you willing to pay a higher price in order to protect the environment?
L2a	To what extent are you willing to pay higher taxes in order to protect the environment?
L3a	To what extent are you willing to reduce your standard of living in order to protect the environment?

Table 2 presents the CFA results for the λ parameter of each potential variable corresponding to each observed variable. All the λ values are standardized. The parameter λ explains the variance, indicating the extent to which an observed variable can reflect the changes in a potential variable (values >0.4 are considered acceptable).

**Table 2:** Values of λ for each potential variable.

**Empathy with nature (EN)**			**Anthropocentrism (AM)**			**Awareness of environmental consequences (AC)**			**Willingness to sacrifice for the environment (WE)**		
	**Estimate**	***p*** **-value**		**Estimate**	***p*** **-value**		**Estimate**	***p*** **-value**		**Estimate**	***p*** **-value**
H3	0.740	0.000	H2	0.640	0.000	P2	0.271	0.000	L1a	0.827	0.000
H5	0.690	0.000	H6	0.567	0.000	P7	0.117	0.000	L2a	0.844	0.000
H13	0.443	0.000	H8	0.665	0.000	P3a	0.296	0.000	L3a	0.570	0.000
			H12	0.697	0.000	P4a	0.428	0.000			
			H14	0.593	0.000	P5a	0.539	0.000			

The designed model's Chi-square = 1,215.296; degrees of freedom = 131; its *p*-value = 0.0000 (< 0.005); root mean square error of the approximation (RMSEA) of the model's goodness-of-fit index (GFI) = 0.067 (< 0.08); Comparative Fit Index (CFI) = 0.832 (>0.80), and the Tucker-Lewis Index (TLI) = 0.804 (>0.90). All of the aforementioned results comply with the standard.

It is important to emphasize that although some components have weighting values that do not appear to be statistically significant, we believe that the questions themselves are closely related to the indicators that we want to measure in this study, and therefore, they are still retained.

### 3.3 Models and statistical analyses

We conducted our analyses in two stages. In the first stage, we ran a structural equation model (SEM). Using Anthropocentrism (AM) and Empathy with Nature (EN) as parallel mediators, we examined their impact on the process from having environmental awareness to being willing to engage in environmental behavior.

To further investigate the relationship between religiosity and awareness of environmental protection and willingness to behave in an environmentally friendly manner, we conducted another multi-cluster analyses. We divided the total sample into three groups: none of religion (*N* = 1,738), Eastern traditional religious belief (*N* = 62), and Western traditional religious belief (*N* = 60), based on people's religious belief categories. Anthropocentrism (AM) and Empathy with Nature (EN) were used as independent variables to examine their effects on environmental awareness and environmental behavior.

In the process of compiling the documents, we also noticed that the traditional primitive religions and folk customs of some regions also served to protect the local environment (Adewoyin et al., 2021; Gairola, 2020; Osemeobo, 1994). Therefore, in designing the grouping, we also included folkloric religious beliefs in the “Eastern traditional religious belief.”

We employed MPLUS 8.0 software to conduct our data analysis using a structural equation model (SEM). Drawing upon relevant studies and theories, we posited a positive relationship between Awareness of Environmental Consequences (AC) and Willingness to Sacrifice for the Environment (WE), implying that an increase in AC would lead to a corresponding increase in WE. Further, we include Empathy with Nature (EN) and Anthropocentrism (AM) as mediator variables in the structural equation model to observe whether they have a significant effect.

Throughout the model fitting process, we will evaluate the adequacy of the model based on various data fitting indicators, such as the χ^2^ test, RMSEA, CFI, and others. In the event of a well-fitting model and significant path coefficients, we can draw meaningful conclusions about the association between variables.

**Figure 1:**
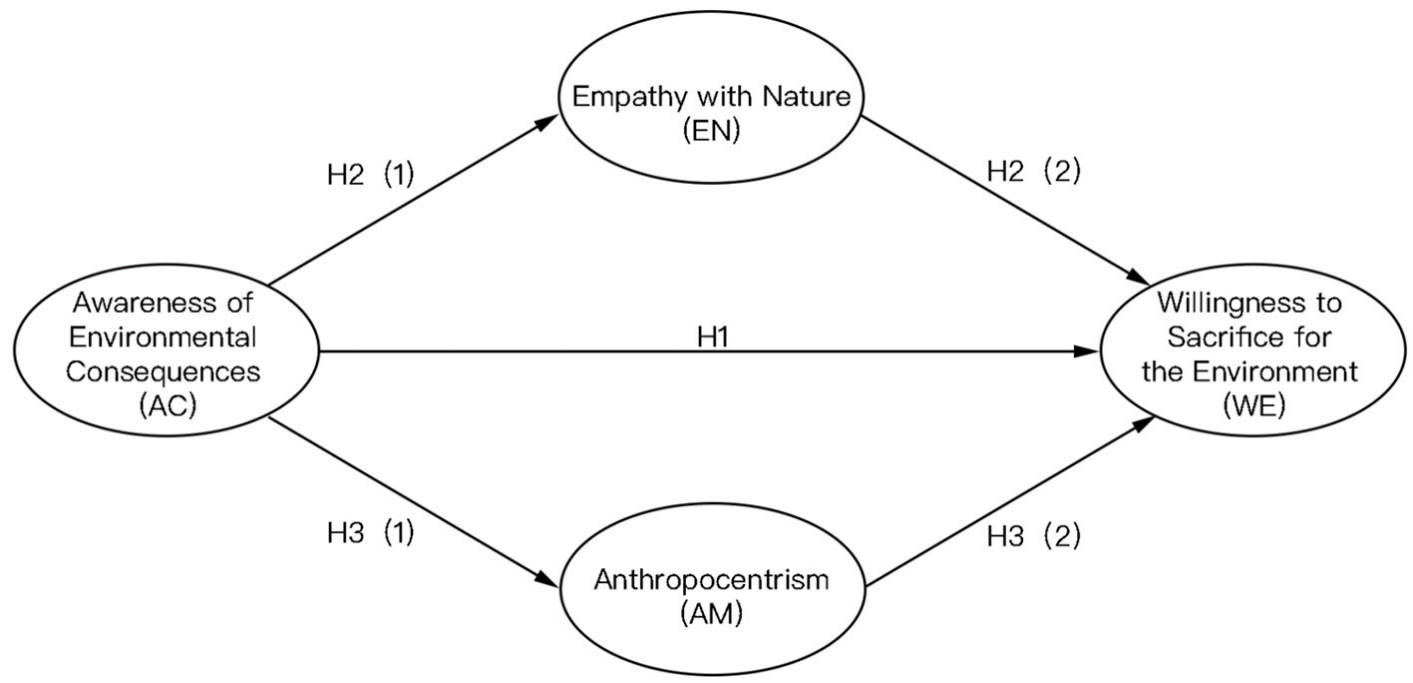
Parallel mediation effect model from AC to WE.

Thus, the research model of this study comprises the following hypotheses (Figure 1):

- *Hypothesis 1 (H1): The awareness of environmental consequences positively influences individuals' willingness to sacrifice for the environment*.
- *Hypothesis 2 (H2): The awareness of environmental further influences individuals' willingness to sacrifice for the environment by affecting empathy with nature*.
- *Hypothesis 3 (H3): The awareness of environmental further influences individuals' willingness to sacrifice for the environment by affecting anthropocentrism*.

**Figure 2:**
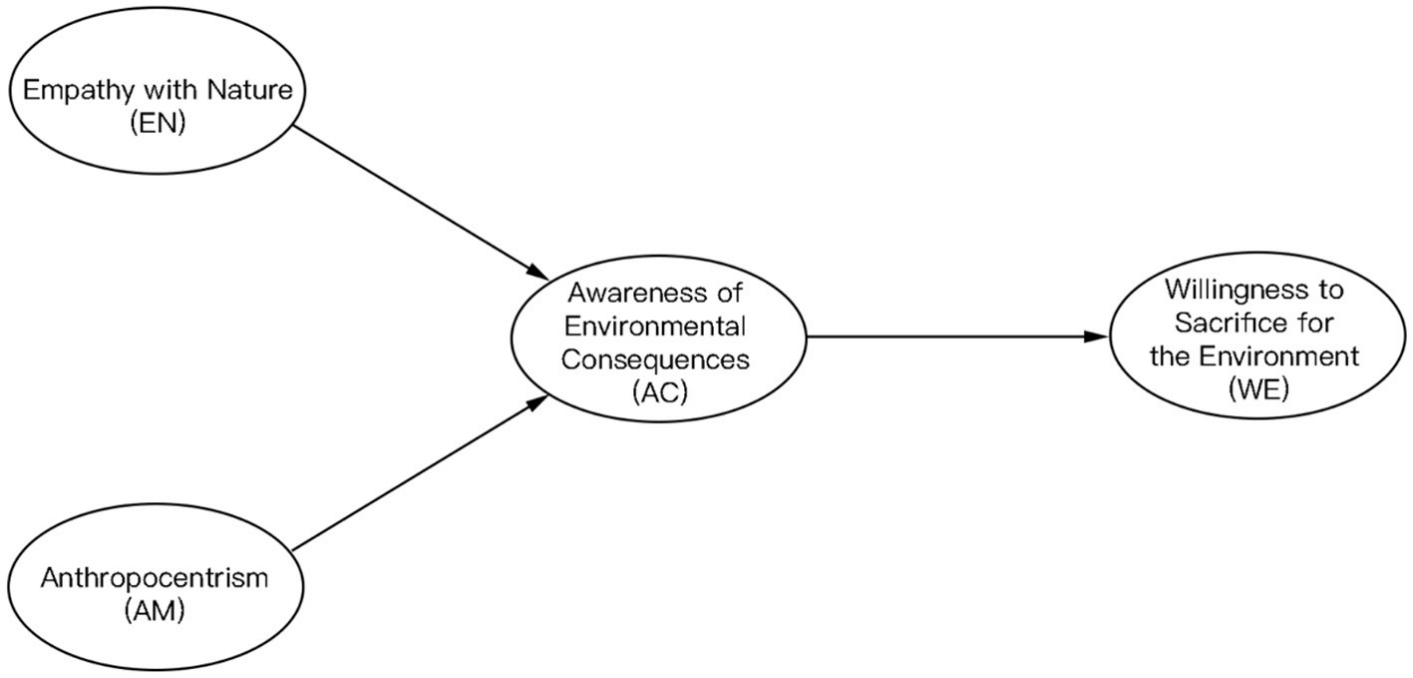
Multi-cluster analysis with dual independent variables.

We further propose the following hypotheses (Figure 2):

- *Hypothesis 4 (H4): Individuals with traditional Eastern religious beliefs exhibit higher levels of environmental consciousness compared to individuals without religious beliefs*.
- *Hypothesis 5 (H5): Individuals with traditional Eastern religious beliefs exhibit higher levels of environmental consciousness compared to those with traditional Western religious beliefs*.

## 4 Results

### 4.1 Hypothesis testing for Model 1

In Model 1, we did not consider the difference in religious beliefs at the moment, and simply examined whether Anthropocentrism (AM) and Empathy with Nature (EN) had a mediation effect on Awareness of Environmental Consequences (AC) and Willingness to Sacrifice for the Environment (WE).

**Figure 3:**
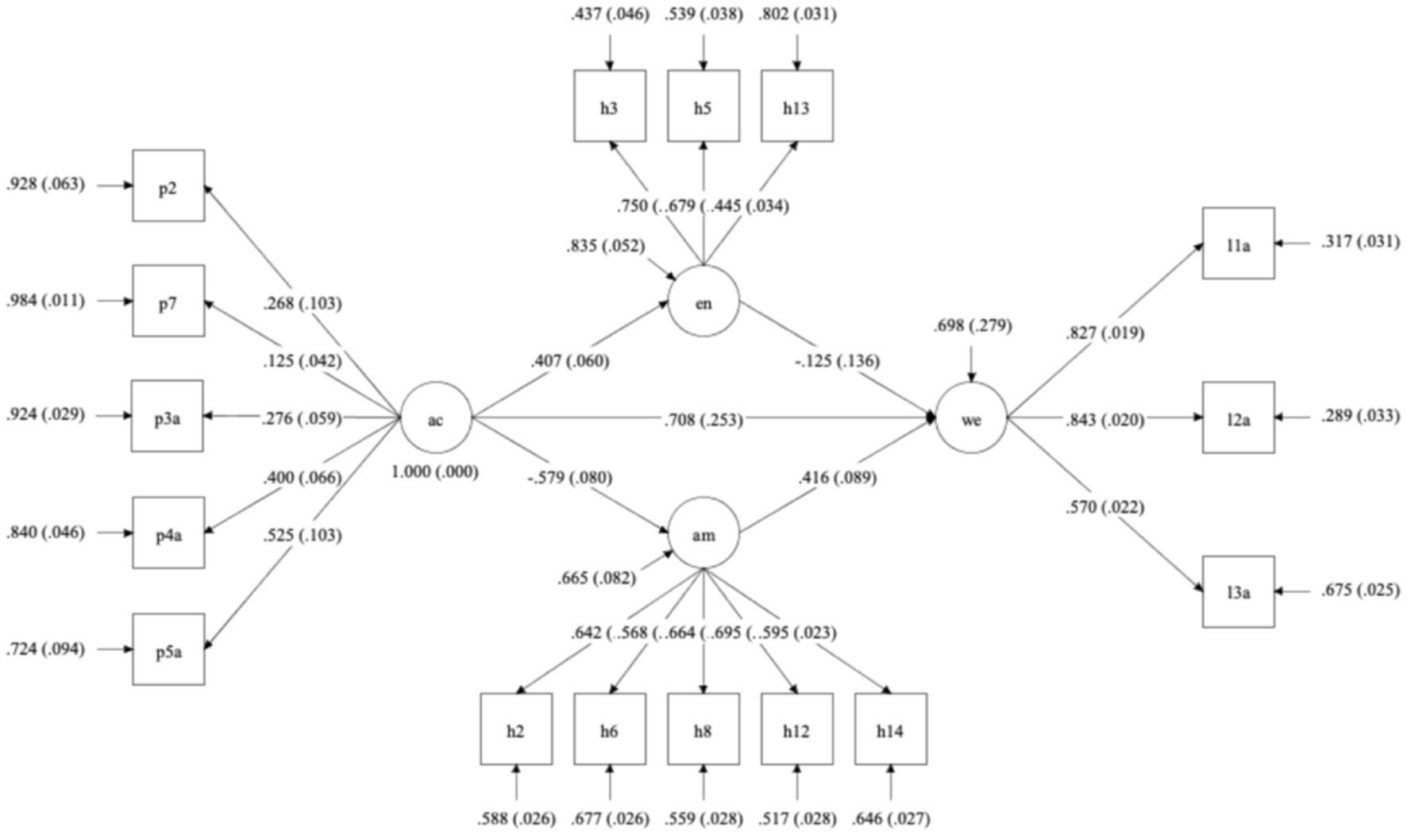
Path diagram about parallel mediation effect model from AC to WE. EN, empathy with nature; AM, anthropocentrism; AC, awareness of environmental consequences; WE, willingness to sacrifice for the environment.

In this model, RMSEA = 0.063 (< 0.08), CFI = 0.881 (>0.80), as shown in Figure 3 thus, the model fit results are acceptable.

In the path analysis, the awareness of environmental consequences has a significant direct effect on willingness to sacrifice for the environment, with a path coefficient of 0.708 (*p* = 0.005). To test the indirect effect, we employed the bootstrap method and calculated a 95% Confidence Interval (CI). If the 95% CI does not include 0, it indicates the presence of an indirect effect (Guo et al., 2018). In conclude, the results of testing the first three hypotheses are presented in Table 3.

**Table 3:** Hypothesis verification.

	**Hypothesis**	**Significance**	**Verification**
H1	The awareness of environmental consequences positively influences individuals' willingness to sacrifice for the environment	*p* = 0.005	Supported
H2(1)	The awareness of environmental has a significant effect on empathy with nature	*p* = 0.000	Supported
H2(2)	Empathy with nature has a significant effect on individuals' willingness to sacrifice for the environment	*p* = 0.360	Not supported
H2	The awareness of environmental further influences individuals' willingness to sacrifice for the environment by affecting the Empathy with nature	*p* = 0.488	Not supported
H3(1)	The awareness of environmental has a significant effect on anthropocentrism	*p* = 0.000	Supported
H3(2)	Anthropocentrism has a significant effect on empathy with nature	*p* = 0.000	Supported
H3	The awareness of environmental further influences individuals' willingness to sacrifice for the environment by affecting the Anthropocentrism	*p* = 0.000	Supported

As shown in Table 4, the indirect effect from the awareness of environmental consequences to the willingness to sacrifice for the environment through anthropocentrism was −0.240 (*p* < 0.001), and the 95% CI did not include 0. The indirect effect from the awareness of environmental consequences to the willingness to sacrifice for the environment through empathy with nature was −0.051 (*p* = 0.488), although the 95% CI did not include 0. This suggests an important mediating role for anthropocentrism and a less pronounced role for natural empathy.

**Table 4:** Indirect effect between peer interaction and PSSE.

**Indirect path**	**β**	**SE**	**95% CI**
AC → EN → WE	−0.051	0.073	(−0.187, −0.002)
AC → AM → WE	−0.240^***^	0.063	(−0.389, −0.167)
Total indirect	−0.291^**^	0.253	(−0.492, −0.178)

^*^p < 0.05, ^**^p < 0.01, ^***^p < 0.001.

The results of this model show that the total indirect effect of the model is negatively correlated, and the total direct effect is positively correlated. This indicates that people who are aware of environmental issues are more inclined to make sacrifices for environmental protection and are willing to prioritize situations where their personal interests are compromised.

However, the results for only one of the two paths are significant and have a negative effect on individuals' willingness to be environmentally friendly. This result nicely supports our hypothesis, indicating that people with anthropocentric tendencies are more reluctant to concede personal benefits to protect the environment. Also, the results for the path through empathy with nature are not significant, indicating that empathy with nature does not affect people's willingness to sacrifice for environmental protection.

### 4.2 Hypothesis testing for Model 2

In Model 2, we constructed a multi-cluster analysis with dual independent variables. In this model, we first divide the total sample into three groups according to different religious affiliation categories: Group 1 = none of religion (*N* = 1,738)/ Group 2 = Eastern traditional religious belief (*N* = 62)/Group 3 = Western traditional religious belief (*N* = 60).

**Figure 4:**
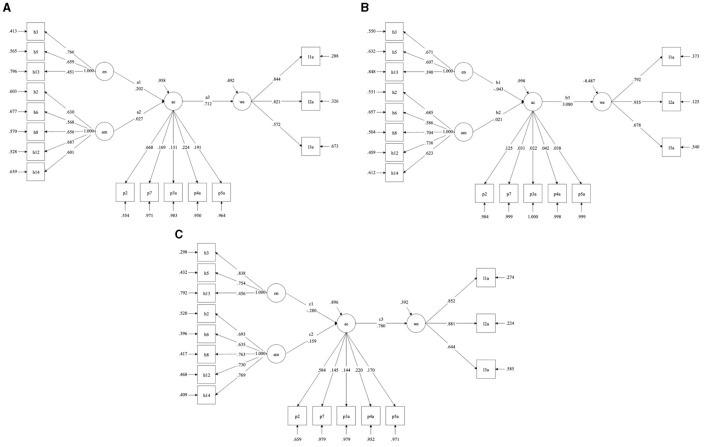
Results under different groups. **(A)** Group1 (none). **(B)** Group2 (Eastern traditional religious belief). **(C)** Group3 (Western traditional religious belief).

Secondly, we include anthropocentrism (AM) and empathy with nature (EN) as independent variables and directly examine their effects on awareness of environmental consequences (AC) and willingness to sacrifice for the environment (WE). The results of the inter-group comparison are provided in detail in Figure 4.

For the model results, we focus on the path differences among the three groups. The detection results are as follows (in Table 5):

**Table 5:** Path difference results.

**Path**	**Estimate**	***p*-Value**
DIF1 (a1–b1)	0.167	0.537
DIF2 (a1–c1)	0.323	0.092
DIF3 (b1–c1)	0.157	0.617
DIF4 (a2–b2)	0.020	0.822
DIF5 (a2–c2)	0.137	0.442
DIF6 (b2–c2)	0.117	0.583
DIF7 (a3–b3)	−22.363	0.000
DIF8 (a3–c3)	−0.247	0.899
DIF9 (b3–c3)	22.116	0.000

On the one hand, the path difference results for DIF7 and DIF9 are significant, indicating that the group with eastern traditional religious beliefs is more environmentally conscious than the group without beliefs and the group with western traditional religious beliefs. This result also successfully verifies our hypotheses 4 and 5.

On the other hand, the direct differences in the other paths were not significant, indicating that there is no statistically significant difference between the effects of anthropocentrism and natural empathy on people under different categories of religiosity.

However, in general, we still believe that religious beliefs have a significant moderating effect on the relationship between awareness of environmental issues and willingness to act in environmental protection.

## 5 Discussion

This study explores the complex relationship between religious beliefs, environmental awareness, and pro-environmental behavior. Our findings provide partial support for the hypotheses, with four of the five hypotheses being supported, and one (Hypothesis 2) showing no statistically significant result. The study confirms that both religious beliefs and the resulting environmental attitudes can influence individual behaviors related to environmental protection. However, the results also reveal some intriguing contradictions, which may be attributed to the unique religious context in Chinese society and the limitations of the research sample.

### 5.1 The effects of anthropocentrism and natural empathy on environmental awareness and willingness to sacrifice

In this study, we used structural equation modeling to test five hypotheses, focusing on the roles of anthropocentrism and natural empathy in shaping people's attitudes and behaviors toward environmental protection. Our results suggest that individuals with Eastern religious beliefs are more likely to make personal sacrifices for environmental protection compared to those without religious beliefs or those adhering to Western religious traditions.

However, the relationship between natural empathy and pro-environmental behaviors is not as clear. While previous research has shown that empathy toward nature positively influences environmental behaviors (Berenguer, 2007, 2010), our study found that natural empathy did not have a statistically significant effect on willingness to protect the environment. Instead, anthropocentrism, or a focus on human-centered concerns, had a stronger negative influence on pro-environmental behaviors. This suggests that while empathy may heighten awareness of environmental issues, it may not be enough to motivate significant behavior changes unless personal interests are at stake. Research has shown that there is often a gap between awareness and the translation of this awareness into concrete actions. In many cases, individual self-interest tends to outweigh moral considerations when it comes to actual behavior. People may recognize the importance of environmental protection, but without direct personal benefits or the perception that their actions will lead to meaningful change, they are less likely to engage in behaviors that require personal sacrifice or effort. This highlights the complexity of pro-environmental behavior, where intrinsic moral values often conflict with immediate self-interests or perceived costs, thus influencing the likelihood of action.

Additionally, while the mediating effect of “natural empathy” was found to be statistically significant at the 95% confidence level, with a confidence interval of (−0.187, 0.002), we must exercise caution in over-interpreting this result. Further research is needed to confirm whether natural empathy truly influences environmental behaviors in the way that previous studies have suggested.

### 5.2 Eastern traditional religious beliefs are more environmentally

Our study also highlights the influence of Eastern religious beliefs on environmental attitudes and behaviors. The long-standing traditions of Confucianism, Buddhism, and Taoism have profoundly shaped Chinese culture, contributing to a worldview that emphasizes harmony between humans and nature. These religions promote the idea that humans are an integral part of the natural world, advocating for respect, stewardship, and sustainable living. Confucianism encourages harmony between humans and the environment (Nuyen, 2008), Buddhism stresses compassion for all living beings (James and Cooper, 2007), and Taoism emphasizes unity between heaven and humanity (Lai et al., 2022).

It is worth noting that Confucianism was not listed as a religious belief in the survey, as it does not follow the same spiritual structure as religions like Buddhism or Taoism. Confucianism promotes a philosophy of “engagement with the world” (入世), encouraging individuals to strive for moral excellence and social responsibility in their everyday lives, rather than advocating for withdrawal from the world or worship of deities. This distinction likely contributes to Confucianism's omission from the list of religious options, despite its pervasive influence on Chinese thought and culture.

Our findings suggest that individuals who adhere to Eastern religious traditions are more likely to engage in pro-environmental behaviors, possibly due to the deep cultural roots of these beliefs. These religious teachings advocate for ecological sustainability, biodiversity preservation, and responsible resource use, which aligns with modern environmental protection efforts. We believe that these Eastern religious values can play a key role in shaping a more symbiotic relationship between humans and nature, contributing to the long-term health of the environment.

In light of this, we encourage religious organizations and leaders to play a proactive role in raising environmental awareness and promoting sustainable practices among their followers. This could provide an important avenue for addressing global environmental challenges, including climate change.

### 5.3 Limitations and directions for future research

Despite the valuable insights gained, this study is not without limitations. One limitation is the sample composition: in Chinese society, where there has been no history of religious state experience, the number of religious adherents is relatively small compared to non-believers. As a result, the subgroup analysis of religious individuals had a smaller sample size, which may have influenced the stability of the results. The classification of Confucianism as a religion presents challenges. Despite its significant cultural influence, Confucianism is often not recognized as a formal religion in China. As a result, many individuals who follow Confucian principles may self-identify as non-religious in surveys, leading to an overrepresentation of non-religious respondents.

Additionally, this study did not directly examine cultural or policy factors, which may also influence pro-environmental behaviors. We recommend future research to explore how cultural and policy factors interact with religious beliefs to shape environmental attitudes and behaviors.

## 6 Conclusion

In conclusion, this study highlights the significant role of religious beliefs—particularly Eastern traditions—in shaping individuals' environmental attitudes and behaviors. Our findings suggest that religious ideologies, especially those rooted in Eastern religions like Buddhism, Taoism, and Confucianism, have a profound influence on promoting pro-environmental actions. However, the study also reveals that anthropocentrism, or the prioritization of human interests, remains a dominant factor when personal stakes are involved, which limits the translation of environmental awareness into action.

## Data Availability

The datasets presented in this study can be found in online repositories. The names of the repository/repositories and accession number(s) can be found below: CGSS 2021 dataset (Chinese General Social Survey, http://www.cnsda.org/index.php?r=projects/view&id=65635422).
